# Adult's Degenerative Scoliosis: Midterm Results of Dynamic Stabilization without Fusion in Elderly Patients—Is It Effective?

**DOI:** 10.1155/2013/365059

**Published:** 2013-05-26

**Authors:** Mario Di Silvestre, Francesco Lolli, Tiziana Greggi, Francesco Vommaro, Andrea Baioni

**Affiliations:** Spine Surgery Department, Istituti Ortopedici Rizzoli, Via Pupilli 1, 40136 Bologna, Italy

## Abstract

*Study Design*. A retrospective study. *Purpose*. Posterolateral fusion with pedicle screw instrumentation used for degenerative lumbar scoliosis can lead to several complications. In elderly patients without sagittal imbalance, dynamic stabilization could represent an option to avoid these adverse events. *Methods*. 57 patients treated by dynamic stabilization without fusion were included. All patients had degenerative lumbar *de novo* scoliosis (average Cobb angle 17.2°), without sagittal imbalance, associated in 52 cases (91%) with vertebral canal stenosis and in 24 (42%) with degenerative spondylolisthesis. Nineteen patients (33%) had previously undergone lumbar spinal surgery. *Results*. At an average followup of 77 months, clinical results improved with statistical significance. Scoliosis Cobb angle was 17.2° (range, 12° to 38°) before surgery and 11.3° (range, 4° to 26°) at last follow-up. In the patients with associated spondylolisthesis, anterior vertebral translation was 19.5% (range, 12% to 27%) before surgery, 16.7% (range, 0% to 25%) after surgery, and 17.5% (range, 0% to 27%) at followup. Complications incidence was low (14%), and few patients required revision surgery (4%). *Conclusions*. In elderly patients with mild degenerative lumbar scoliosis without sagittal imbalance, pedicle screw-based dynamic stabilization is an effective option, with low complications incidence, granting curve stabilization during time and satisfying clinical results.

## 1. Introduction

Degenerative lumbar scoliosis, also described as* de novo *or “primary degenerative scoliosis” [1] is a frequent disease. Its incidence is reported to be from 6% to 68% [2–5] and increases with age [6]. These curves are located at thoracolumbar or lumbar level and need to be distinguished from degenerated preexisting idiopathic scoliosis; in fact, *de novo* scoliosis is developing after skeletal maturity without previous history of scoliosis. A recent prospective study [3] investigated 60 adults aged 50–84 years, without previous scoliosis. within 12 years, 22 cases (36.7%) developed *de novo* scoliosis with a mean angle of 13°. A previous study reported a similar incidence: Robin et al. [7] followed 160 adults with a straight spine for more than 7 years and found 55 cases of *de novo* scoliosis (34.4%). Decreased bone density was initially considered to be the cause of *de novo* lumbar scoliosis [2]. At present, asymmetric degenerative changes of the disc, vertebral body wedging, and facet joint arthritis are held to be the predominant causes [1, 3, 7–9], disc degeneration appearing to be the starting point [3, 8]. Lumbar *de novo* scoliosis is frequently associated with degenerative spondylolisthesis and stenosis [6, 10, 11].

The surgical treatment of these deformities included more often a posterolateral fusion with pedicle screw instrumentation in addition to decompression of neural elements [1, 12–15]. In most series, the incidence of complications is high [1, 13–15]. The impact of different factors on the complications rate remains unclear and there are conflicting results in the literature [13–21]. However, older age (over 65 years), medical comorbidities, increased blood loss, and number of levels fused seem to play an important role. Among these, excessive intraoperative blood loss seems to be the most significant risk factor for early perioperative complications [14]. Accordingly, in elderly patients, the surgery should be the least aggressive possible, and the length of the surgical procedure should be considered very carefully [20]. A surgical treatment based on decompression alone presented poor results, related to progression of symptoms and deformity [22]. At the same time, adding an arthrodesis to the decompression procedure increases the operative time and blood loss and consequently can increase the complications rate [13, 16, 21].

The use of dynamic stabilization without fusion can represent an option for treatment of mild degenerative lumbar scoliosis without sagittal imbalance. In a previous study [23], we analyzed the outcomes of dynamic stabilization for these deformities, using Dynesys implants (Zimmer Spine, Minneapolis, MN) as an alternative to fusion in elderly patients. The purpose of the present paper is to assess the midterm results of a larger series of patients over 65 years, in order to determine complications and to evaluate clinical outcomes.

## 2. Materials and Methods

A retrospective data base review was performed to identify all patients affected by degenerative lumbar “*de novo*” scoliosis (Aebi's classification type I [1]), who had been surgically treated by dynamic fixation (Dynesys system) without fusion at our department between January 2002 and December 2006. 

Inclusion criteria were (1) minimum age at surgery of 65 years; (2) Cobb angle more than 10° before surgery; (3) no improvement after conservative treatment; (4) minimum-5-year followup. 

Exclusion criteria were (1) fixed sagittal imbalance; (2) scoliosis Cobb angle more than 40° before surgery; (3) previous lumbar fusion or stabilization surgery.

An independent spine surgeon reviewed all the selected patients' medical records and X-rays. Inpatient and outpatient charts were used for collecting demographic data, preoperative data (location of pain, neurologic symptoms, and previous surgeries), perioperative data (blood loss, surgical duration, hospital stay, and any medical and surgical-related complication), and postoperative data, including revision surgeries. 

### 2.1. Questionnaires

Clinical outcome was assessed by means of the Oswestry Disability Index (ODI), Roland Morris Disability Questionnaire (RMDQ), and separate visual analog scales (VAS) for back and leg pain, completed by patients preoperatively, in the early postoperative period and at last followup. Radiographic evaluation included preoperative CT and MRI of the lumbar spine, as well as pre-operative, postoperative and followup standing plain radiographs. Overall measures from the radiographs included Cobb angle of the lumbar curve, lumbar lordosis (T12-S1) and thoracolumbar junction alignment (T10-L2), apical vertebral lateral displacement, and anterior vertebral translation measurements for spondylolisthesis. Instrumentation loosening or breakage and degenerative alterations of adjacent levels were also investigated. 

### 2.2. Statistical Evaluation

The clinical and radiologic results were analyzed using *t*-test. Results are expressed as the mean (range), with a *P* value < 0.05 considered as being statistically significant. 

### 2.3. Surgical Treatment

All surgeries were performed by four experienced spine surgeons of our department. Preventive antibiotics were routinely started 12 hours before surgery and continued for an average of 9 days (range, 8 to 11 days). The patients were treated under general anesthesia in the prone position. 

Initially, in cases with associated stenosis of the vertebral canal, patients' hips were flexed at an angle of 90° to facilitate decompression of the stenotic levels. Stenosis was treated by laminectomy: the decompression was extended to the lateral recess, and foraminotomy was performed without interrupting the isthmus. 

After decompression, the patients' position was modified to obtain the maximum lumbar lordosis, and stabilization was performed. 

**Figure 1:**
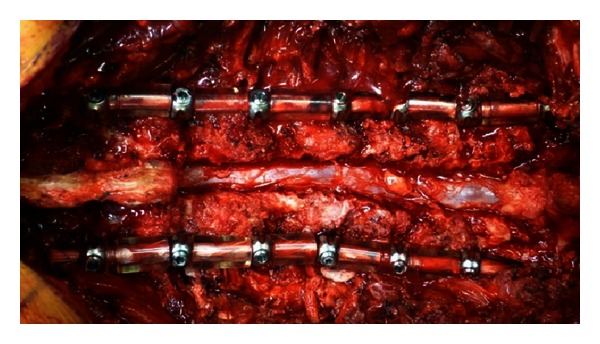
Multisegmental dynamic stabilization with decompressive laminectomy.

Dynesys implants were used for dynamic fixation. Dynesys implants consist of titanium alloy pedicle screws (Protasul 100), polyethylene-terephthalate cords (Sulene-PET), and polycarbonate urethane spacers (Sulene-PCU), which fit between the pedicle screw heads (Figure 1). The pedicle screws used in lumbar, thoracolumbar vertebrae, and in the sacrum were 7.2 mm diameter screws. The pedicle entry point was lateral, at the basis of the transverse process. The screws were inserted as deep as possible. So as not to compromise the bone purchase of the screws, given their conical core, we avoided removing and reinserting them in the same hole. Each of the polycarbonate urethane spacers was cut to the desired length and threated with a polyester cord, which was stretched between and fixed to two adjacent screw heads. Larger spacers were used on the concave side and shorter on the convex side of the scoliosis curve.

Redon drains were applied and maintained for a mean of 3.7 days (range: 3 to 4 days). 

## 3. Results

### 3.1. Preoperative Data

One hundred twenty-five consecutive patients were assessed for eligibility: 68 were excluded. Reasons were incomplete radiographic documentation (*n* = 4), previous spinal fusion or instrumentation (*n* = 16), scoliosis Cobb angle >40° (*n* = 20), fixed sagittal imbalance (*n* = 25), and age <60 years (*n* = 3).

A total of 57 patients were included in the study and reviewed at a mean followup time of 77 months (range: 61 to 91 months) (Table 1). There were 12 men (22%) and 45 women (78%), with a mean age of 68.4 years (range: 66 to 78). At the time of surgery, all 57 patients reported leg pain; 47 (82%) also had neurogenic claudication, and 42 (73%) had back pain. All patients had failed to respond to conservative treatment conducted for at least 12 months.

Average BMI was 26.4 (range: 21 to 36). There were 1.8 ± 0.7 comorbidities per patient, including diabetes mellitus in 25 patients, heart disease in 12, arterial hypertension in 38, liver disease in 13, and pulmonary disease in 16 cases. All patients had degenerative lumbar *de novo* scoliosis (with an average Cobb angle of 17.2°), associated in 52 cases (91%) with vertebral canal stenosis. Twenty-four patients (42%) also presented with degenerative spondylolisthesis, at L2-L3 level in 2 cases, at L3-L4 in 12 cases, at L4-L5 in 7 cases, and at L5-S1 in 3 cases (3 patients had spondylolisthesis at two levels): the mean slippage was 18.9% (range: 12% to 27%). Nineteen patients (33%) had previously undergone lumbar spinal surgery, including decompressions and/or discectomies (seven patients had had 2 previous operation, five had had 2 operations, and 2 patients had had three). 

### 3.2. Perioperative Data

**Figure 2:**
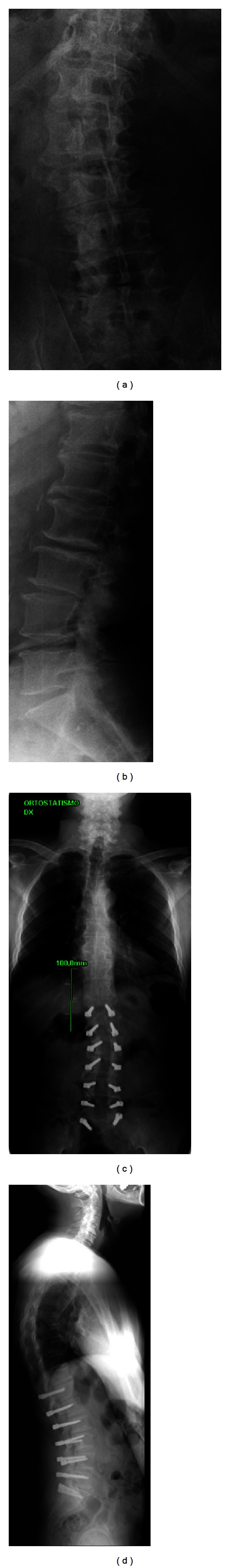
A 73-year-old woman. Degenerative lumbar scoliosis with good sagittal balance ((a)-(b)), associated with stenosis of the vertebral canal. Treatment: T12-S1 dynamic stabilization and decompressive laminectomy. Five-year postoperative radiographs showing stable scoliosis correction with maintained sagittal balance ((c)-(d)).

**Figure 3:**
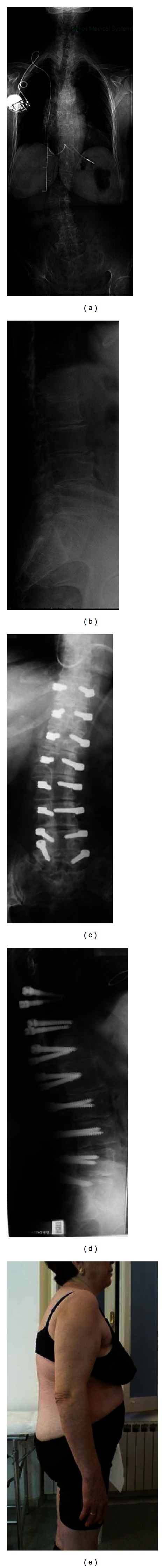
A 71-year-old woman. Degenerative lumbar scoliosis associated with stenosis of the vertebral canal: good sagittal balance ((a), (b)). Treatment: T11-S1 dynamic fixation and decompressive laminectomy. Six-year and 9 months postoperative radiographs showing stable scoliosis correction with maintained sagittal balance ((c), (d), and (e)).

All patients had dynamic stabilization without fusion (Figures 2 and 3). Three levels were stabilized in 31 patients (54%: L1-L4 in 6, L2-L5 in 16, and L3-S1 in 9), four levels in 11 patients (19%: L1-L5 in 7, L2-S1 in 4), five levels in 5 cases (8%: T12-L5 in 3, L1-S1 in 2), six levels in 8 patients (15%: T12-S1), and seven levels in 2 patients (4%: T11-S1).

In 52 patients (91%), the stabilization was combined with decompressive laminectomy of 2 levels in 7 cases (14%: L2-L3 in 3, L3-L4 in and 2, L4-L5 in 2), of 3 levels in 14 cases (27%: L2-L4 in 7, L3-L5 in 7), of 4 levels in 13 cases (25%: L2-L5 in 6, L3-S1 in 7 cases), of 5 levels in 10 cases (19%: L1-L5 in 6, L2-S1 in 4), and of 6 levels in 8 cases (15%: T12-L5 in 6, L1-S1 in 2). If present, the associated spondylolisthesis was always included in the stabilization construct. 

Mean operating time was 170 minutes (range: 120 to 210 minutes), mean hospital stay was 6.8 days (range: 6 to 9 days) and mean blood loss was 650 cc (range: 200 to 700 cc). Patients were returned to the upright position at 2.6 days postoperatively (range, 2 to 4 days), with a lumbar orthosis, which was prescribed for 1 month.

### 3.3. Clinical Outcome (See Table 2)

The mean preoperative ODI score was 51.6% (range, 28 to 80), mean postoperative score was 27.2 (range, 0 to 66), and the final followup score was 27.7 (range, 0 to 70) (*P* < 0.05), with a mean final improvement of 51.6% (range, 12% to 100%) (*P* < 0.05).

The mean preoperative RMDQ score was 12.4 of 24 (range, 7 to 22), mean postoperative score was 6.0 (range: 0 to 19), and final followup score was 6.3 (range, 0 to 20) (*P* < 0.05), with a mean final improvement of 58.8% (range: 9.1% to 100%) (*P* < 0.05). 

The mean leg pain VAS decreased from a preoperative score of 67.5 (range: 30 to 100) to a mean postoperative score of 40.1 (range: 2 to 90) and to a score of 41.6 (range: 2 to 90) at the last followup (*P* < 0.05), with a mean final improvement of 51.1% (range, 10% to 96.4%) (*P* < 0.05). The mean back pain VAS decreased from a preoperative score of 66.7 (range: 30 to 100) to a postoperative score of 33.1 (range: 2 to 75) and to a score of 33.8 (range: 2 to 79) at last followup (*P* < 0.05), with a mean final improvement of 57.4% (range: 20% to 97.0%) (*P* < 0.05). 

### 3.4. Radiologic Outcome (See Table 3)

The average scoliosis Cobb angle was 17.2° (range: 12° to 38°) before surgery, 11.0° (range, 4° to 26°) after surgery and remained stable, and 11.3° (range: 4° to 26°), at last followup (*P* < 0.05). Lumbar lordosis was −30.6° (range: 3° to −39°) before surgery, −36.8° (range: −12° to −57°) after surgery, and −35.8° (range: −10° to −55°) at last followup (*P* < 0.05), with a mean final improvement of 6.4% (range: 0% to 17%) (*P* < 0.05). 

Thoracolumbar junction alignment (TLJA) (T10-L2) was −2.8° before surgery (range: −25° to 23°), −0.2° (range: −18° to 25°) after surgery, and −0.4° (range: −18° to 25°) at last followup.

Apical vertebra lateral listhesis (AVLL) was 1.2 cm (range: 0.2 to 2.0 cm) before surgery, 0.8 cm (range: 0.2 to 1.1 cm) after surgery, and 0.8 cm (range: 0.3 to 1.2 cm) at last followup (*P* < 0.05), with a mean final correction of 30.7% (range: 0% to 44.4%) (*P* < 0.05). 

In the patients with associated spondylolisthesis, anterior vertebral translation was 19.5% (range: 12% to 27%) before surgery, 16.7% (range: 0% to 25%) after surgery, and 17.5% (range: 0% to 27%) at followup (*P* < 0.05), for a 14.9% mean correction (range: 0 to 100%) (*P* < 0.05). 

### 3.5. Complications (See Table 4)

No neurological complications were observed in any patient: 8 overall complications (14%) occurred. 

Six patients (10%) had minor complications. These included two cases of ileus (4%) and two urinary tract infection (4%), which resolved after medical treatment. Another patient (2%) had transient postoperative delirium, which spontaneously resolved after 3 days. One patient (2%) developed dyspnea after surgery, requiring 5 days of recovery in the Intensive Care Unit for complete resolution. 

Two patients (4%) had major complications that required revision surgery. One patient (2%) developed severe postoperatative sciatica, resistant to medication without neurological deficit, due to a misplaced screw on L5; revision surgery for replacement of the screw was performed 5 days after the first operation, with complete resolution of the sciatica. Another patient (2%) developed persistent leg pain, resistant to medication without neurological deficit, 28 months after surgery, due to disc degeneration at the lower junctional level; revision surgery was performed 32 months after the first operation, with decompression and extension of fixation from L5 to S1. 

No screw loosening or breakage was observed at followup. However, asymptomatic radiolucent lines up to 2 mm around the thread of pedicle screws in the sacrum without screw loosening were found in 5 patients (9%) at last followup. 

## 4. Discussion

The surgical treatment of degenerative lumbar scoliosis in elderly patients presents demanding aspects. The main goals of surgery are pain relief and improvement in quality of life. Some correction of the deformity is desirable, but this is not the most important issue and it is essential to limit the aggressiveness of the surgical procedure as much as possible [10]. Posterolateral fusion with pedicle screw instrumentation in addition to laminectomy [10, 13–15] is the most commonly used procedure. Unfortunately, a high incidence of complications has been reported in older patients [14, 15, 18–20]. Notably, age has been correlated with an increased incidence of complications, with a 20% rate of major complications over 80 years of age [18]. Furthermore, excessive blood loss and the number of levels fused have been found to be associated with higher complication rates [14].

Less invasive than posterior fusion, pedicle screw-based dynamic stabilization without arthrodesis might be a useful alternative in elderly patients with mild degenerative lumbar scoliosis without sagittal imbalance. Previously, our series of degenerative scoliosis patients often associated with lumbar stenosis has shown that it can prevent progression of scoliosis and postoperative instability, even after laminectomy [23]. In that report, operative duration time was short, blood loss was reduced, and there was no screw loosening or breakage at followup. The present study confirms at midterm followup these results with limited blood loss and short operative time. Moreover, dynamic fixation provided substantial stability by preserving against further scoliosis progression or translation of associated spondylolisthesis, despite use of decompressive laminectomy. By applying asymmetric spacers, larger on the concave side and shorter on the convex side of scoliosis, it was possible to obtain a mild reduction of the scoliosis Cobb angle, albeit less than with fusion constructs, reported in the literature [13–15]. There was no case of screw loosening or breakage during followup. In five of the patients (9%), asymptomatic radiolucent lines up to 2 mm did appear around the thread of pedicle screws in S1 at last X-rays control; however, it was not observed a screw mobilization or a loss of scoliosis correction and the patients were asymptomatic, so we did not classify these cases as “unstable”. The overall complication rate was low (14%) with an even markedly lower incidence of major complications (14%). 

A frequent complication observed in elderly patients after posterior fusion is adjacent segment disease, which generally occurs proximal to posterior instrumentation and has been reported primarily after short lumbar fusion [15]. Proximal adjacent disease appears to develop more frequently when stopping fusion from T11 to L1 compared with extending it to T10 [14, 24]. In older patients, the advantage for a “short” posterior fusion is obvious, even if the instrumentation should not stop at a junctional zone or adjacent to a rotatory subluxation, spondylolisthesis, or a segment with significant spinal stenosis, because this may lead to spinal instability. In a recent study, Cho et al. [15] compared the results of short posterior fusion, within the deformity, versus long fusion, extended above the upper end vertebra, for degenerative lumbar scoliosis in patients whose mean age was 65.5 years. In this series, there was a trade-off in complications between short fusion and long fusion; whereas all cases of proximal adjacent segment disease developed in the short fusion group, long fusion induced excessive intraoperative blood loss, which was closely related to the development of perioperative complications.

In our series, elderly patients received a short instrumentation, extended up to T11 at most. Only one patient (2%) required subsequent surgery for adjacent segment deasese and a distal junctional disc degeneration 32 months after surgery. At present, there is no consensus on whether or not dynamic instrumentation protects adjacent levels more than fusion. A study concluded that dynamic stabilization can prevent degeneration of the adjacent segment [25]. However, the results of the study of Schnake et al. [26] after Dynesys instrumentation in cases with degenerative spondylolisthesis did not support this theory. The authors found signs of adjacent degeneration in 29% of the patients after 2 years. Although longer followup studies are necessary for definitive conclusions, the theoretical protective effect of dynamic stabilization against adjacent segment degeneration is consistent with our findings, with a 5-year minimum followup. 

In different series [13–15], posterior fusion obtained a significative scoliosis correction. In our series there was a scoliosis stabilization at a followup of more than 5 years. However, in *de novo* scoliosis patients the goal of treatment is less for the amount of correction of the curve than its stability over time. The same could be said for final lumbar lordosis; dynamic fixation maintained a stable and satisfying lumbar lordosis at followup. The patient's position on the operating table was always assessed to maintain or to increase the lumbar lordosis. All cases included in this study presented preoperatively a satisfying sagittal balance. In cases of sagittal imbalance, it is very difficult to achieve normal lumbar lordosis by dynamic stabilization or posterior fusion alone. Different surgical techniques such as corrective osteotomy should be considered preoperatively in these patients.

Finally, it's important to underline that, at followup (Table 2) dynamic fixation achieved clinically significant improvement in ODI, RMDQ, and VAS scores. 

## 5. Conclusions

The present series must be interpreted in the context of its limitations (the retrospective nature of the review and the fact that patients were not randomized). However, this series of patients is consecutive and they received surgical treatment in the same institution.

In elderly patients with mild degenerative lumbar scoliosis without sagittal imbalance, pedicle screw-based dynamic stabilization permitted to maintain a satisfying balanced spine at follow-up: this procedure resulted less invasive with short operative duration and limited blood loss and low adverse event rates. 

Dynamic fixation achieved scoliosis curve stabilization, at an average followup of more than 5 years. Furthermore, functional outcomes resulted were satisfying at last control.

## Figures and Tables

**Table 1:** Demographic data.

Parameters	Value
Age (yrs)	68.4
Female gender (%)	78%
Comorbidities	1.8 ± 0.7
Deg. spondylolisthesis	42%
Stenosis	91%
Prev. spinal surgery	33%
Leg pain	100%
Back pain	73%
Claudicatio	82%

**Table 2:** Clinical outcome.

	Preop	FU	% corr.	*P *value
ODI	51.6 (28 to 80)	27.7 (0 to 70)	51.6 (12 to 100)	<0.05
RMDQ	12.4 (7 to 22)	6.3 (0 to 20)	58.8 (9.1 to 100)	<0.05
VAS “leg score”	67.5 (30 to 100)	41.6 (2 to 90)	51.1 (10 to 96.4)	<0.05
VAS “back score”	66.7 (30 to 100)	33.8 (2 to 79)	57.4 (20 to 97)	<0.05

Mean value (range: minimum to maximum).

**Table 3:** Radiologic outcome*.

	Preop	FU	% corr	*P* value
Scoliosis (°)	17.2° (12 to 38)	11.3° (4 to 26)	37.3% (13.3 to 61.5)	<0.05
Lordosis	−30.6° (3 to −39)	−35.8° (−10 to −55)	6.4% (0 to 17)	<0.05
TLJA	−2.8° (−25 to 23)	−0.4° (−18 to 25)	n.a.	n.a.
AVLL (cm)	1.2 (0.2 to 2)	0.8 (0.3 to 1.2)	30.7 (0 to 44.4)	<0.05
AVT (%)	19.5% (12 to 27)	17.5% (0 to 27)	14.9% (0 to 100)	<0.05

Mean value (range, minimum to maximum).

TLJA: thoracolumbar junction alignment (T10-L2).

AVLL: apical vertebra lateral listhesis.

AVT: anterior vertebral translation.

n.a.: not available.

**Table 4:** Complications.

Complications	Percentage
Overall	**8 (14%)**

Minor	**6 (10%)**
Ileus	2 (4%)
Urinary tract infection	2 (4%)
Transient delirium	1 (2%)
Dyspnea	1 (2%)

Major	**2 (4%)**
Misplaced screw	1 (2%)
Lower junctional disc degeneration	1 (2%)
